# Crystal structures, electron spin resonance, and thermogravimetric analysis of three mixed-valence copper cyanide polymers

**DOI:** 10.1107/S2053229624003371

**Published:** 2024-05-01

**Authors:** Peter W. R. Corfield, Ahmed Elsayed, Tristan DaCunha, Christopher Bender

**Affiliations:** a Department of Chemistry and Biochemistry, Fordham University, 441 East Fordham Road, Bronx, NY 10458, USA; Universidade Federal de Minas Gerais, Brazil

**Keywords:** crystal structure, mixed valence, copper cyanide, ethano­lamine, propano­lamine, polymeric network, electron spin resonance, ESR, thermogravimetric analysis, TGA

## Abstract

The crystal structures of three mixed-valence copper cyanide alkanolamine polymers are presented, together with thermogravimetric analysis (TGA) and electron spin resonance (ESR) data. In all three structures, a Cu^II^ moiety on a crystallographic center of symmetry is coordinated by two alkanolamines and links two Cu^I^CN chains *via* cyanide bridging groups to form diperiodic sheets. The sheets are linked together by cuprophilic Cu^I^–Cu^I^ inter­actions to form a three-dimensional network. In poly[bis­(μ-3-amino­propano­lato)tetra-μ-cyan­ido-dicopper(I)dicopper(II)], [Cu_4_(CN)_4_(C_3_H_8_NO)_2_]_*n*_, **1**, propano­lamine bases have lost their hydroxyl H atoms and coordinate as chelates to two Cu^II^ atoms to form a dimeric Cu^II^ moiety bridged by the O atoms of the bases with Cu^II^ atoms in square-planar coordination. The ESR spectrum is very broad, indicating exchange between the two Cu^II^ centers. In poly[bis­(2-amino­pro­pan­ol)tetra-μ-cyanido-dicopper(I)copper(II)], [Cu_3_(CN)_4_(C_3_H_9_NO)_2_]_*n*_, **2**, and poly[bis­(2-amino­ethanol)tetra-μ-cyanido-dicopper(I)copper(II)], [Cu_3_(CN)_4_(CH_7_NO)_2_]_*n*_, **3**, a single Cu^II^ atom links the Cu^I^CN chains together *via* CN bridges. The chelating alkanolamines are not ionized, and the OH groups form rather long bonds in the axial positions of the octa­hedrally coordinated Cu^II^ atoms. The coordination geometries of Cu^II^ in **2** and **3** are almost identical, except that the Cu—O distances are longer in **2** than in **3**, which may explain their somewhat different ESR spectra. Thermal decom­position in **2** and **3**, but not in **1**, begins with the loss of HCN(g), and this can be correlated with the presence of OH protons on the ligands in **2** and **3**, which are not present in **1**.

## Abstract 2

The crystal structures of three mixed-valence copper cyanide polymers with similar polymeric CuCN networks are described, together with the results of electron spin resonance and thermogravimetric analyses.

## Chemical context

Polymeric CuCN com­pounds with organic ligands have continued to excite inter­est in light of their varied structures (Pike, 2012 ▸), the magnetic exchange or photoluminesence exhibited by many of them, and other potentially useful physical properties (Lim *et al.*, 2008 ▸). Many hundreds of crystal structures are now known (*e.g.* Nicholas *et al.*, 2019 ▸; Xu *et al.*, 2019 ▸; Etaiw *et al.*, 2016 ▸). One class of such polymers com­prises anionic CuCN frameworks with guest cations providing charge neutrality and we have made systematic studies of such com­pounds containing cations derived from amines and ethano­lamines (Koenigsmann *et al.*, 2020 ▸; Corfield *et al.*, 2022 ▸). Mixed-valence CuCN polymers containing bases coordinated to the Cu^II^ atoms are also well known (Liu *et al.*, 2017 ▸; Qin *et al.*, 2016 ▸), though fewer in number than the Cu^I^CN com­plexes. Such networks would be neutral not anionic, and might therefore be capable of crystallizing with neutral mol­ecules as guests. We have made studies of several such com­plexes involving di­amines (Corfield *et al.*, 2016 ▸), but until recently we were less successful at isolating crystalline com­plexes of mixed-valence CuCN networks involving N-sub­stituted ethano­lamines. In the present article, we de­scribe the isolation and structural characterization of crystals of three such mixed-valence CuCN networks, along with powder ESR data and thermogravimetric analyses: poly[bis­(μ-3-amino­propano­lato)tetra-μ-cyanido-dicopper(I)dicopper(II)], **1** (Scheme 1), with the base propano­lamine, coordinated to Cu^II^ as an alkoxide; poly[bis­(2-amino­pro­pan­ol)tetra-μ-cy­anido-di­copper(I)copper(II)], **2** (Scheme 2), with the base 2-am­ino­pro­pan-1-ol; and poly[bis­(2-amino­ethanol)tetra-μ-cyanido-di­copper(I)copper(II)], **3** (Scheme 3), with the base ethan­o­lamine. 
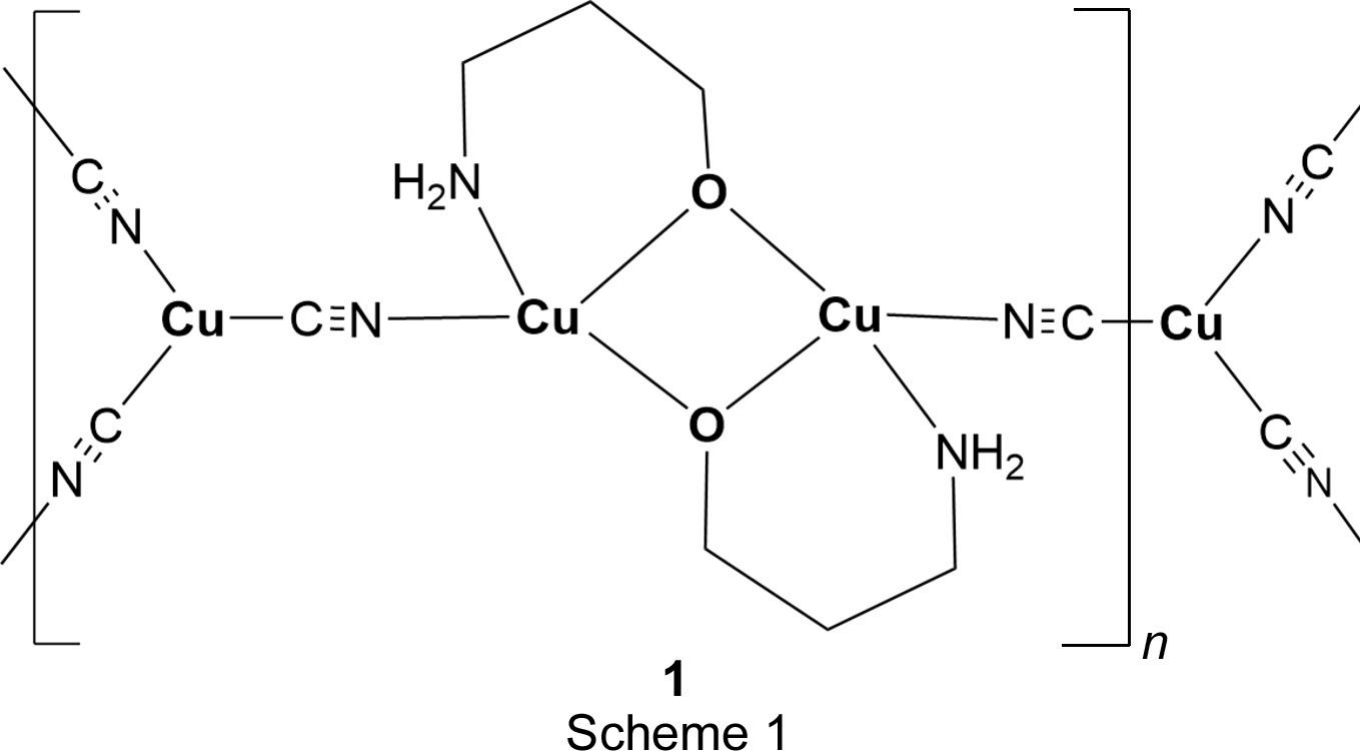
 
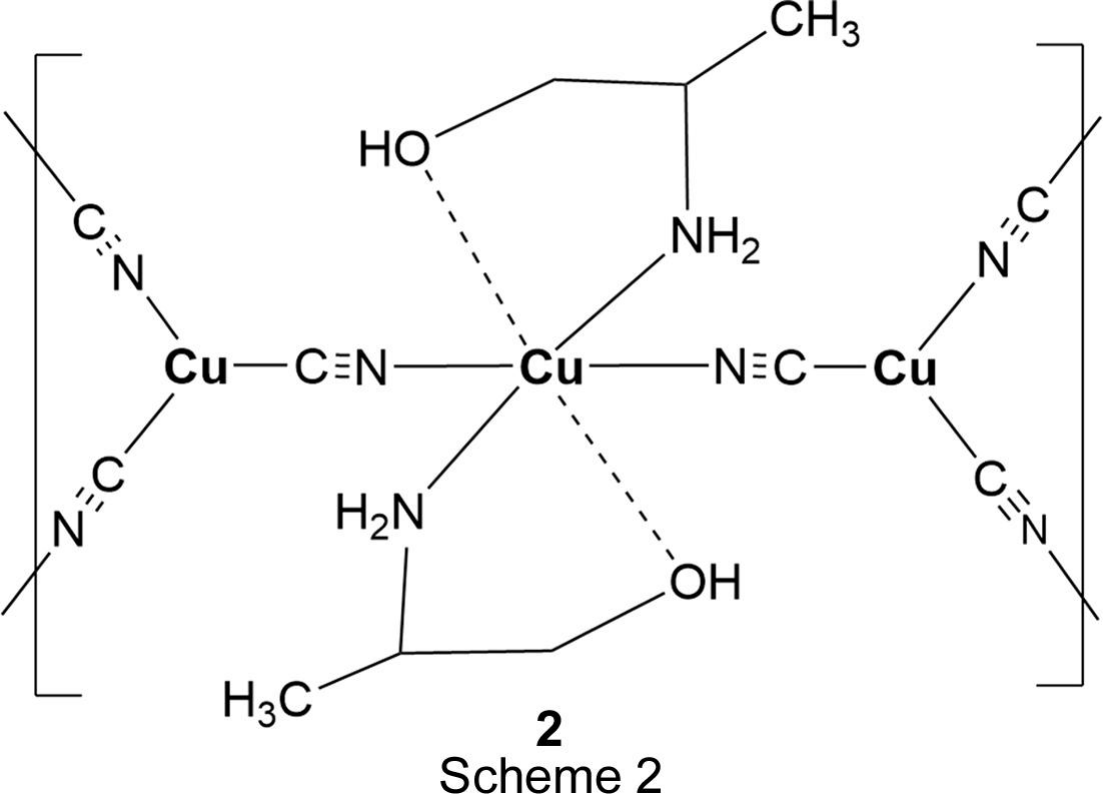


## Experimental

### Synthesis and crystallization

Syntheses were carried out by air oxidation of solutions containing NaCN/CuCN in the presence of the amine ligand. Results varied from batch to batch. Specific details of representative syntheses follow.

For the preparation of **1**, 17 mmol (0.833 g) of NaCN were dissolved in 25 ml of distilled water and 15 mmol (1.343 g) of CuCN were added and the mixture stirred and filtered, as not quite all of the CuCN had dissolved. Then 35 mmol of 3-am­inopro­pan-1-ol (2.629 g) were added with stirring, and the colorless mixture was covered. Red crystals separated after about two weeks (yield: 0.393 g, or 21%, based upon Cu). IR spectra (cm^−1^): 2124 (*s*), 2135 (*s*) (CN stretch); 3255 (*s*), 3298 (*s*) (N—H stretch); 3452 (*m*) (O—H stretch, broad, probably due to moisture contaminant). Preparations under similar conditions did not always produce homogeneous samples, but crystals of **1** were usually present.

For the preparation of **2**, 20 mmol (0.995 g) of NaCN were dissolved in 20 ml of distilled water and 10 mmol (0.893 g) of CuCN were added. The mixture was stirred until a clear solution was obtained. 20 mmol (1.502 g) of 2-aminopro­pan-1-ol were added and the mixture stirred. After about three months, 38 mg of a brown product com­posed of gold–brown plates were obtained. Based upon a mol­ecular formula of Cu_3_(CN)_4_ *L*_2_, where *L* = 2-aminopro­pan-1-ol, this corresponds to a percentage yield of 2.6%. IR spectra (cm^−1^): 2104 (*s*), 2116 (*s*) (CN stretch); 3255 (*m*), 3319 (*m*), 3347 (*w*) (N—H stretch); 3506 (*m*), 3543 (*m*) (O—H stretch). We were surprised to also obtain 184 mg of a crystalline material that gave the same IR spectrum in a separate synthesis designed to give a Cu^I^CN com­plex only. In this case, we used the same procedure, with 5.0 mmol of CuCN instead of 10 mmol, but the base was neutralized before addition to the aqueous NaCN/CuCN mixture, which we presumed would hinder air oxidation of Cu^I^. We do not have an explanation for this synthesis when so many other attempts had been unsuccessful. 
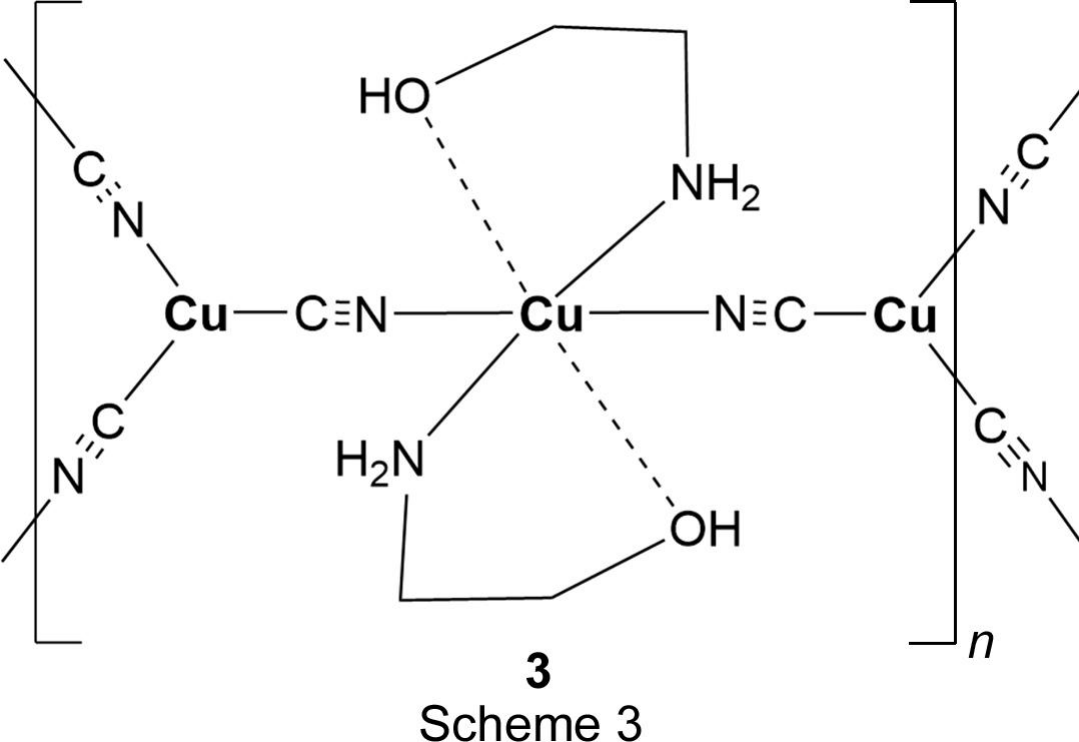


For the preparation of **3**, 39 mmol (1.898 g) of NaCN were dissolved in 8 ml of distilled water and 23 mmol (2.080 g) of CuCN were added, and the mixture stirred until a clear solution was obtained. 21 mmol (1.293 g) of 2-amino­ethanol in 3 ml water were added, and the pale-green mixture stirred. After 5 d, 223 mg of black crystals were obtained, corresponding to a 6.9% yield based on Cu. IR spectra (cm^−1^): 2118 (*s*), 2130 (*s*) (CN stretch); 3266 (*m*), 3233 (*m*) (N—H stretch); 3524 (*m*) (O—H stretch).

### Refinement

Crystal data, data collection and structure refinement details are summarized in Table 1 ▸. For structures **2** and **3**, the tensor analysis in *XABS2* (Parkin *et al.*, 1995 ▸) was used to improve the absorption correction, leading to a somewhat less noisy final difference Fourier map. In all structures, C—H protons were restrained to the expected geometry, with C—H distances of 0.97 Å, while positional coordinates for N—H and O—H protons were refined. For C—H hydrogens, the *U*_iso_(H) values were constrained to 1.2*U*_eq_ of the adjacent atoms for **1**, and 1.5*U*_eq_ for **2** and **3**. In **1**, the low-angle 020 reflection was omitted as it was partially obscured by the beamstop. For **3**, data sets from two crystals were merged using *SORTAV* (Blessing, 1989 ▸). The size of the second smaller crystal was 0.15 × 0.10 × 0.06 mm.

In all three structures, CN group occupancies were refined, initially set with 50% disorder. The occupancies for all CN groups bound to Cu^II^ clearly indicated that these groups are *N*-bonded to Cu^II^, as expected, while the refined occupancies for the CN groups linking Cu^I^ atoms were not significantly different from 50%. Thus, only the Cu^I^ CN groups were modeled as disordered and were fixed at 50% in all structures.

In **1**, atoms C12, C13, and C14 of the chelate ring Cu2/O11/C12–C14/N15/Cu2 were treated as disordered above and below the central plane of the six-membered ring, and the *A*/*B* occupancies refined to 74 (2) and 26 (2)%. In both **2** and **3**, the chelating ethano­lamine ligands were modeled as disordered between λ and δ conformations. All the ligand atoms were counted as disordered, except that the tightly bound NH_2_ atoms of the two disorder mates were constrained in both structures to the same positions, whereas the more loosely bound OH atoms were allowed to refine independently, along with the ligand C atoms, with constraints on the displacement parameters for the O atoms. The *A*/*B* occupancies refined to 53.4 (9) and 46.6 (9)% for **2**, and to 64.3 (16) and 35.7 (16)% for **3**.

## Results and discussion

### Structural commentary

**Figure 1:**
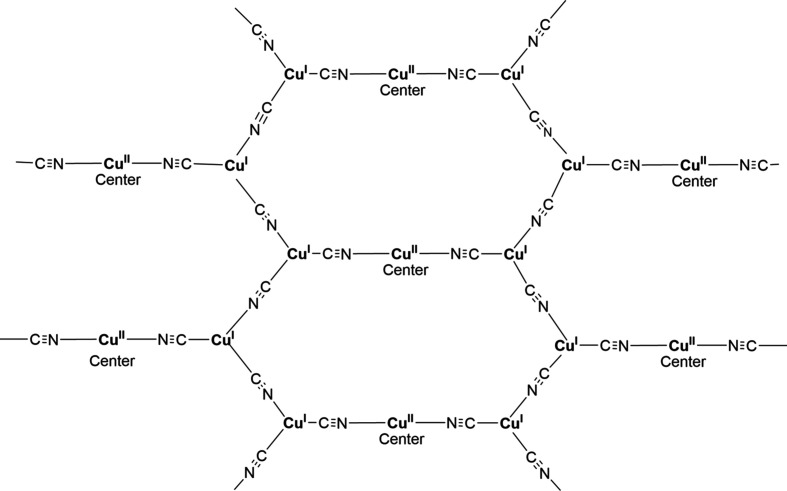
General scheme for the title com­pounds.

In each of the three title structures, a Cu^II^ moiety coordinated by chelated alkanolamine bases links zigzag Cu^I^CN chains into a diperiodic network *via* bridging CN groups, with the Cu^II^ moiety situated at a crystallographic center of symmetry, as seen in the general scheme (Fig. 1 ▸). In every case, the C atoms of the chelated rings are disordered. For convenience, we have labeled Cu^I^ atoms as Cu1 and Cu^II^ atoms as Cu2 in the discussion that follows.

**Figure 2:**
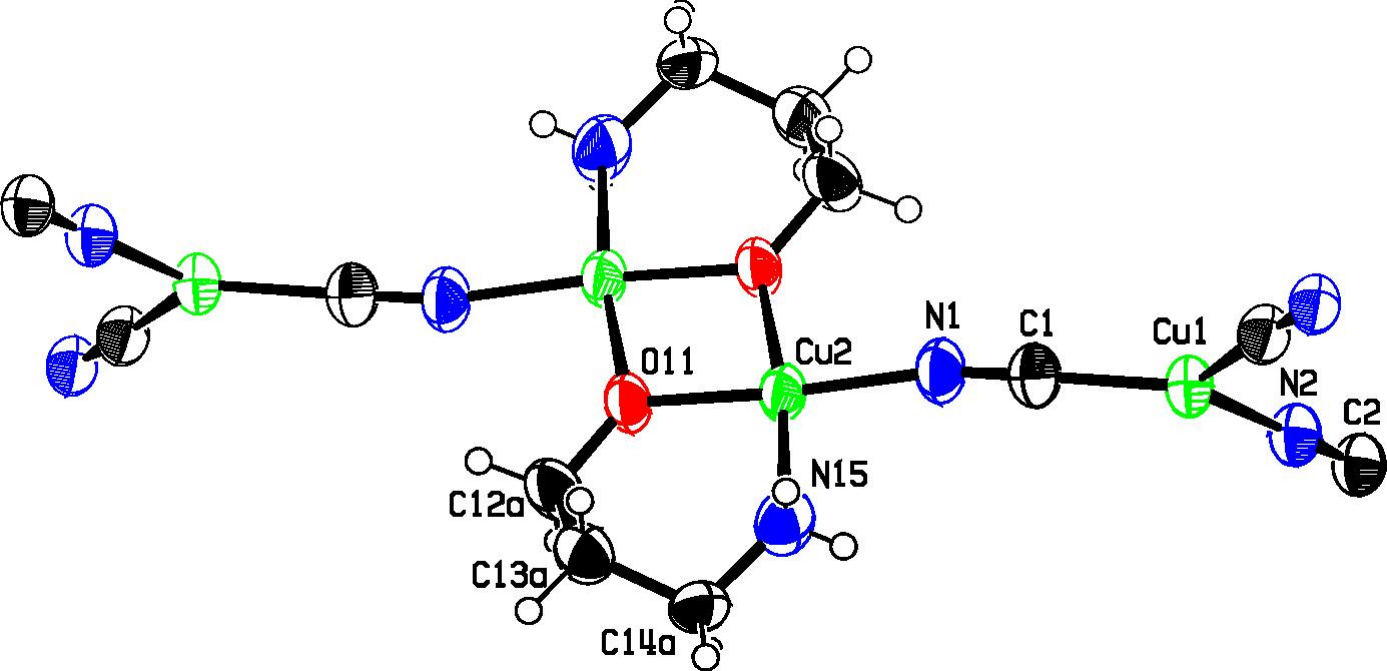
The structure of **1**, showing the atom numbering and 50% displacement ellipsoids, with H atoms depicted as small spheres. Only the major-disorder com­ponent for the chelate ring is shown. Cu atoms are shown in green, N atoms in blue, O atoms in red, and C and H atoms in black. The asymmetric unit is highlighted in bold.

In the structure of **1**, shown in Fig. 2 ▸, the propano­lamine bases have lost their hydroxyl H atoms, and coordinate as chelates to two Cu2 atoms to form a dimeric Cu^II^ moiety bridged by the O atoms of the bases to form a central four-atom ring. The eight central Cu, N, and O atoms of the dimeric moiety are roughly coplanar, with r.m.s. deviation from the best plane of 0.023 Å. The Cu2 atoms are in a distorted square-planar coordination, with the O—Cu—O angle in the four-membered ring equal to 76.91 (8)° and the other bond angles ranging from 91.15 (11) to 96.64 (9)°. The fourth ligand to each Cu2 atom is a CN group which bridges to a Cu1 atom bonded to two other CN groups. The bond angles at Cu1 are 111.63 (10), 122.02 (10), and 125.89 (10)°, with the smallest angle *trans* to the CN bridging to Cu2.

**Figure 3:**
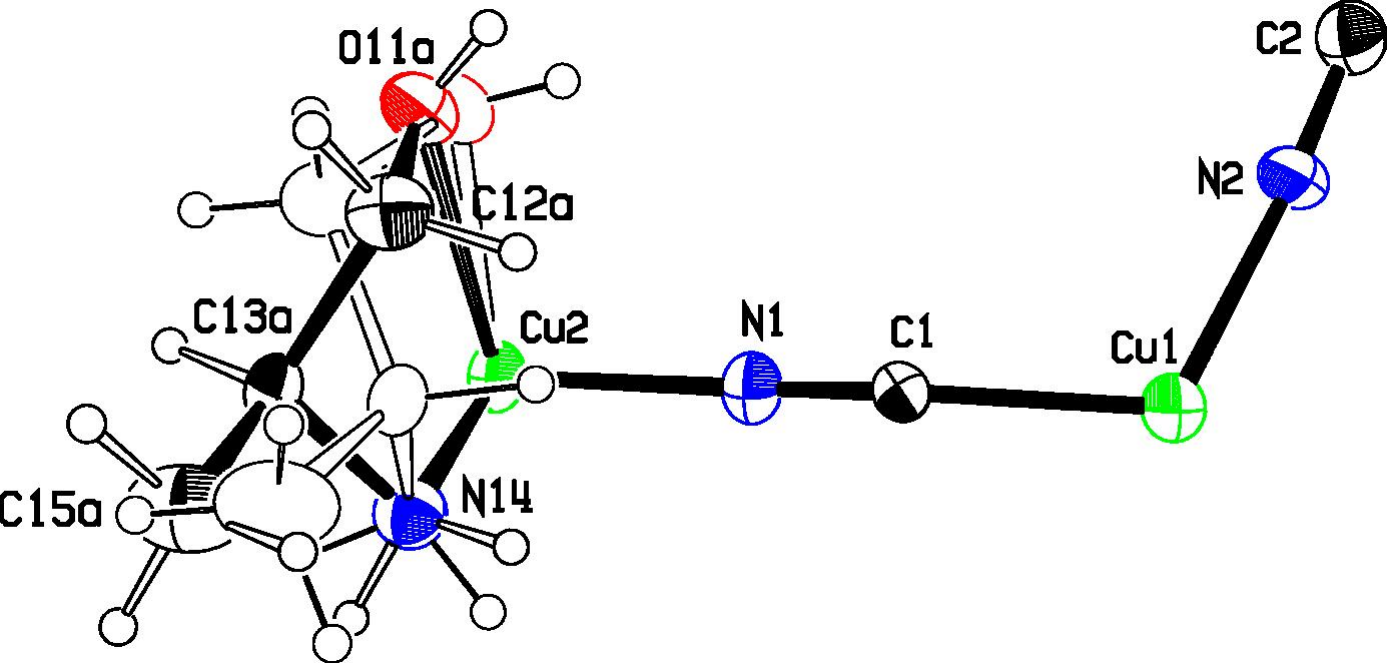
The asymmetric unit of **2**, showing the atom numbering and 50% displacement ellipsoids. The colors are as in Fig. 2 ▸.

**Figure 5:**
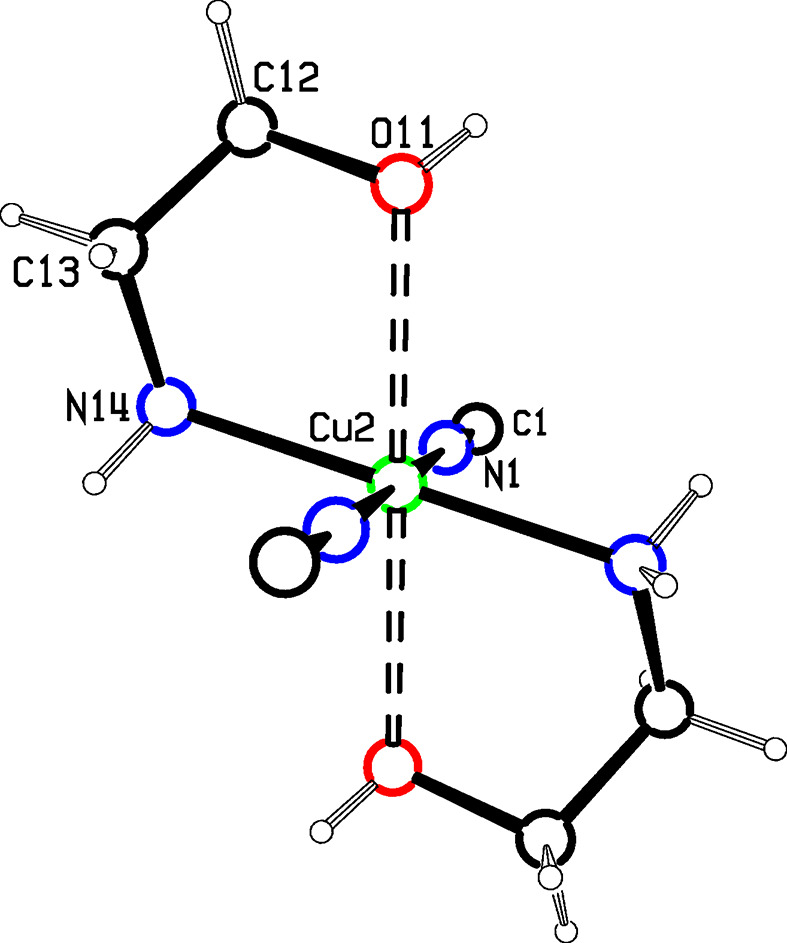
The Cu^II^ coordination in **3**. The coordination in **2** is the same, with a methyl group added to position C12.

Figs. 3 ▸ and 4 ▸ show the asymmetric units for structures **2** and **3**, where single Cu2 atoms play the role of the dimeric moieties in **1** in linking the Cu1 chains into diperiodic structures. Cu2 atoms are octa­hedrally coordinated by two cyanide groups and by two chelating ligands, which are disordered between λ and δ conformations. The coordination is illustrated in Fig. 5 ▸, and com­parisons of bond lengths and angles with **1** are given in Table 2 ▸. The coordination geometries are almost identical for com­pounds **2** and **3**. In these two structures, the ligands have not lost their OH protons and the Cu—O distances are much longer than in **1** where the bonding is to an alkoxide. The Cu—NH_2_ and Cu—NC bonds are also slightly longer in **2** and **3** than in **1**. The bond angles at Cu1 in **2** range from 113.4 (2) to 121.8 (2)°, while in **3** they range from 118.33 (12) to 120.78 (11)°. In both cases, the largest angle is *trans* to the CN bridging to Cu^II^, in contrast to **1**, where there is a larger deviation of angles from 120° and the smallest angle is *trans* to the CN—Cu2 bridge.

The structure of **3** has been reported previously (Jin *et al.,* 2006 ▸), but was redone in our laboratory for consistency. In Jin *et al.*, the space group was given as *C*2/*m*. We chose *C*2/*c*, as in all crystals that we have studied, reflections with *k* + *l* odd are present, though systematically weak; in *C*2/*m*, *k* + *l* reflections would be absent, leading to a slightly different structure. Jin *et al.* did not record the presence of the OH proton, so that database searches based upon ethano­lamine do not lead to their structure.

### Supra­molecular features

**Figure 6:**
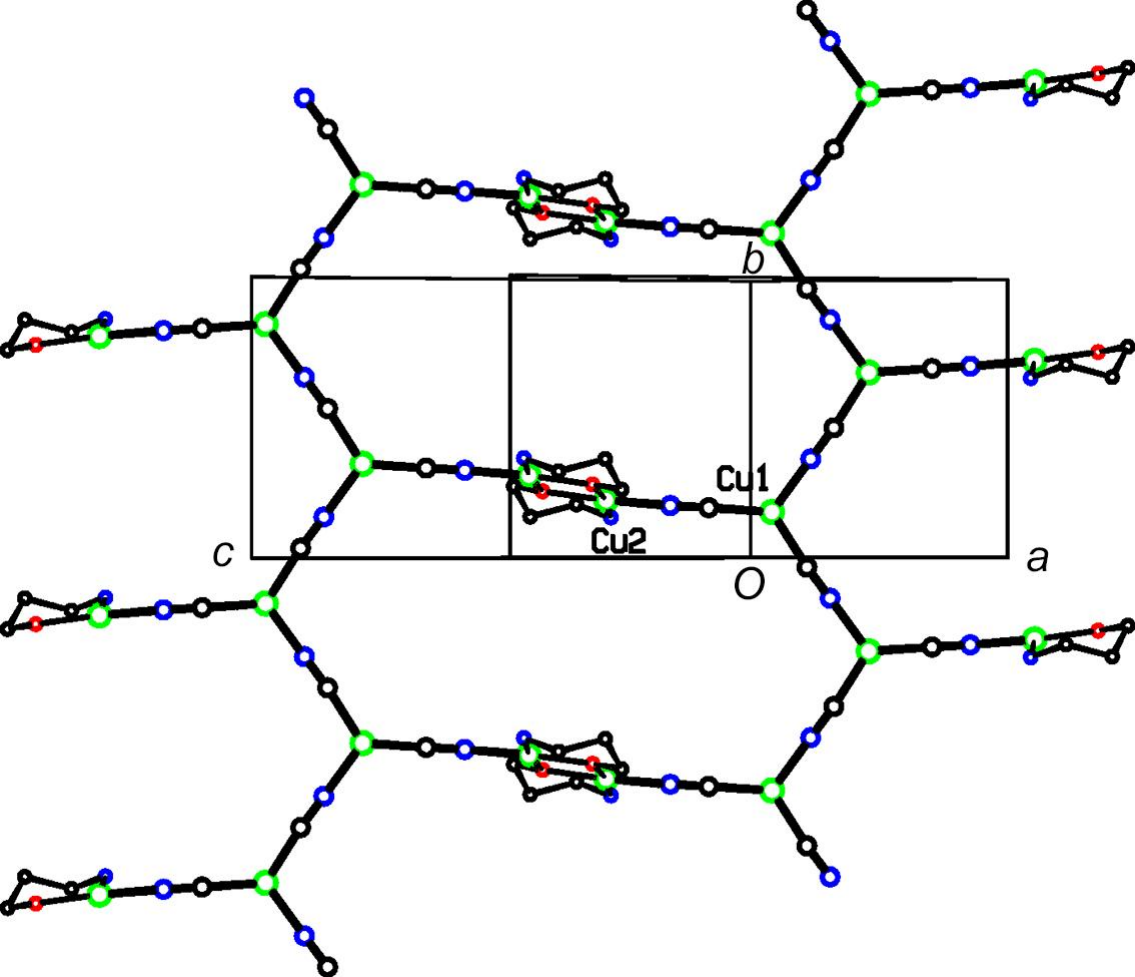
A diperiodic sheet in **1**. The colors are as in Fig. 2 ▸.

**Figure 7:**
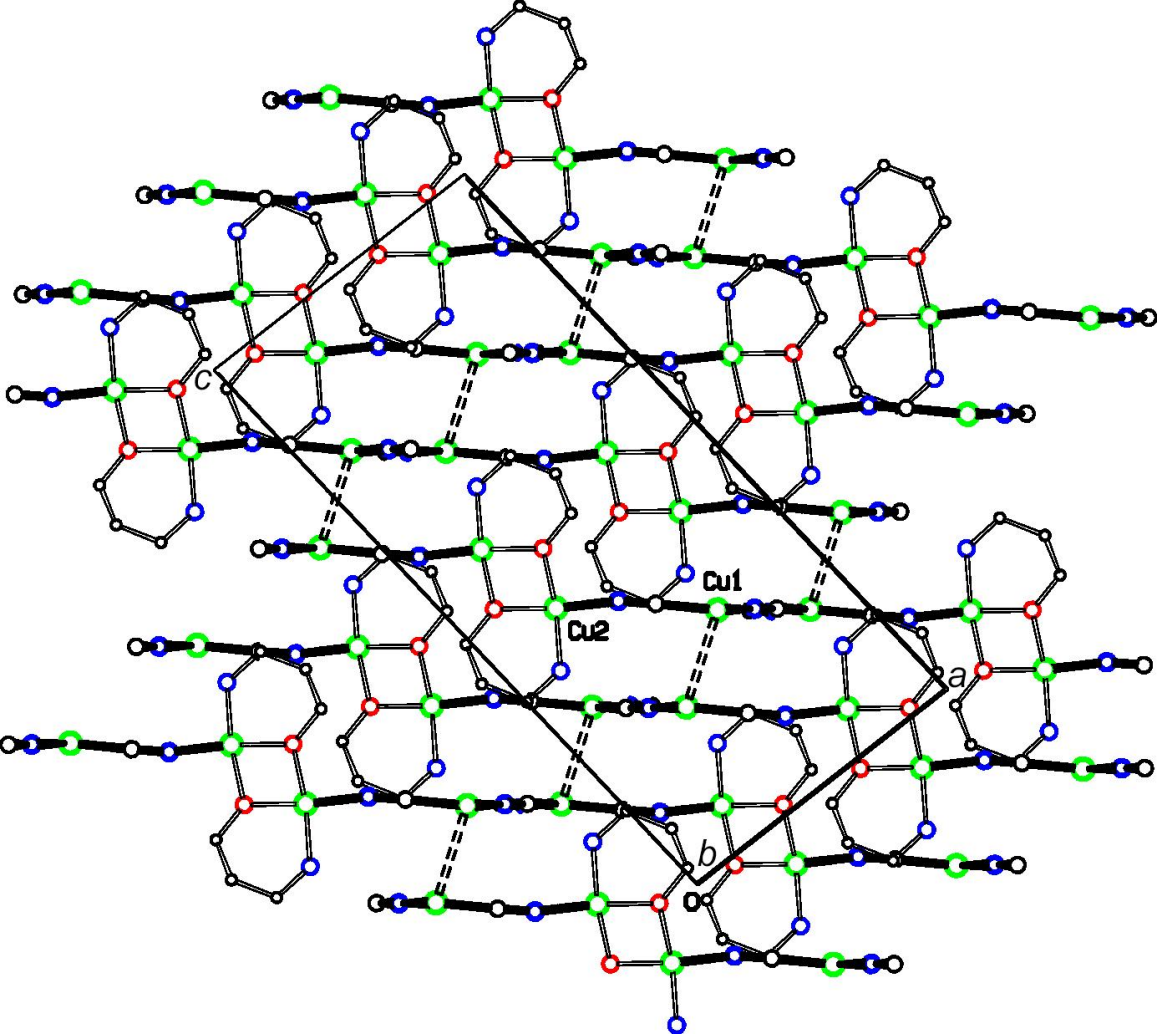
The packing in **1**. The sheets shown in Fig. 6 ▸ are viewed edge on. Putative cuprophilic bonds are shown as dashed double lines.

In **1**, the dimeric Cu2 moieties bridge monoperiodic Cu^I^CN chains to form diperiodic networks parallel to (102), as shown in Figs. 6 ▸ and 7 ▸. The Cu^I^CN zigzag chains extend in the direction of the *b* axis, out of the plane of Fig. 7 ▸. The plane of the Cu^I^CN chain network makes an angle of 85.5 (1)° with the eight-atom dimeric Cu2 plane. Cu1 atoms from neighboring sheets are within 3.103 (1) Å of one another, in roughly axial positions with regard to their trigonal planar coordination. Also, the Cu1 atom lies 0.075 (2) Å out of the plane of its three ligands, which brings it closer to the neighboring Cu1 atom in the neighboring sheet. This weak cuprophilic inter­action links the sheets into a triperiodic network, and is shown as a dashed line in Fig. 7 ▸. Atom H14*B* is found on the other side of the Cu1 coordination plane, at 3.10 Å from Cu1. Otherwise, there are no other short inter­molecular contacts of note in this structure.

**Figure 8:**
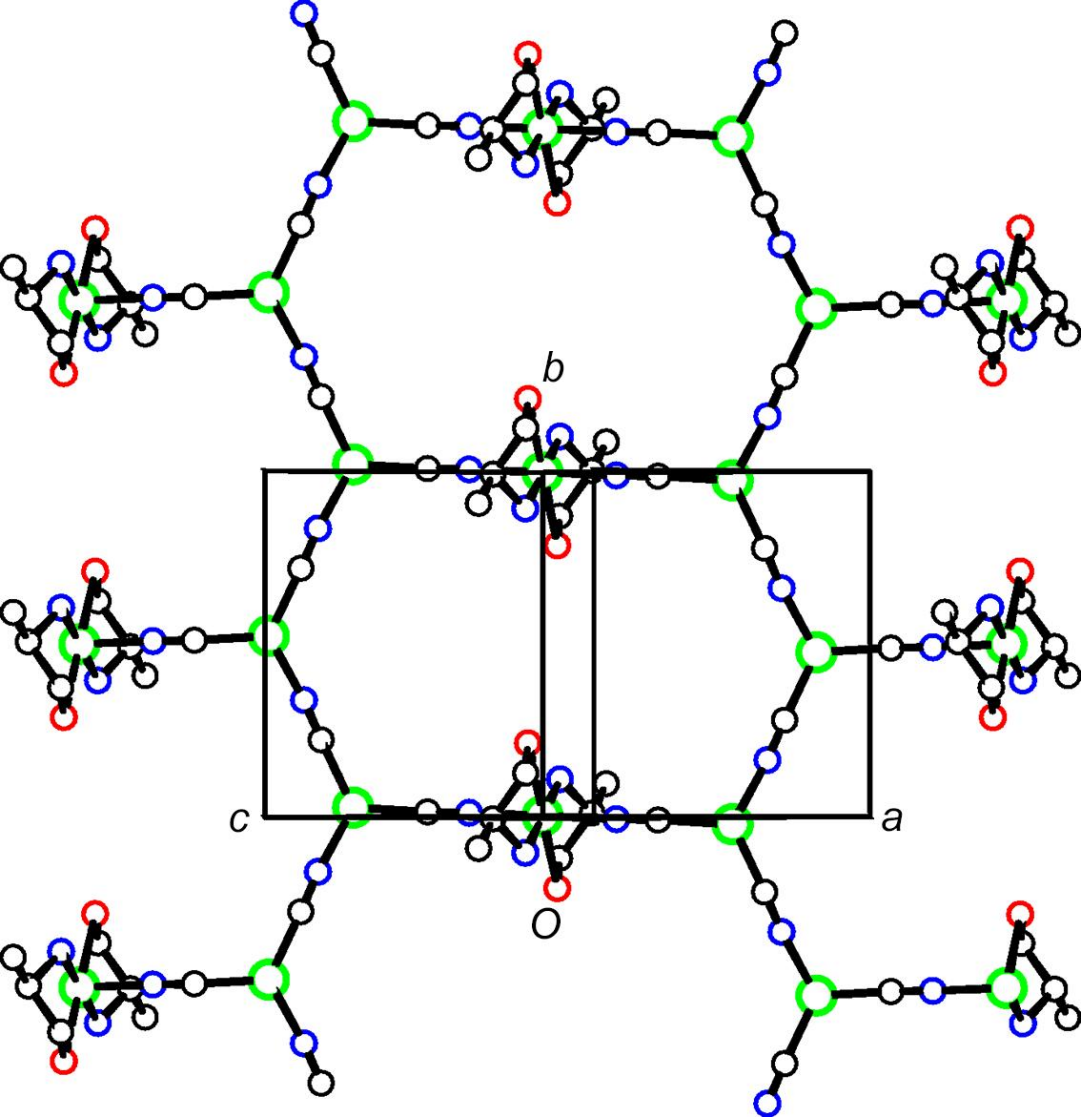
A diperiodic sheet in **2**. The colors are as in Fig. 2 ▸.

**Figure 10:**
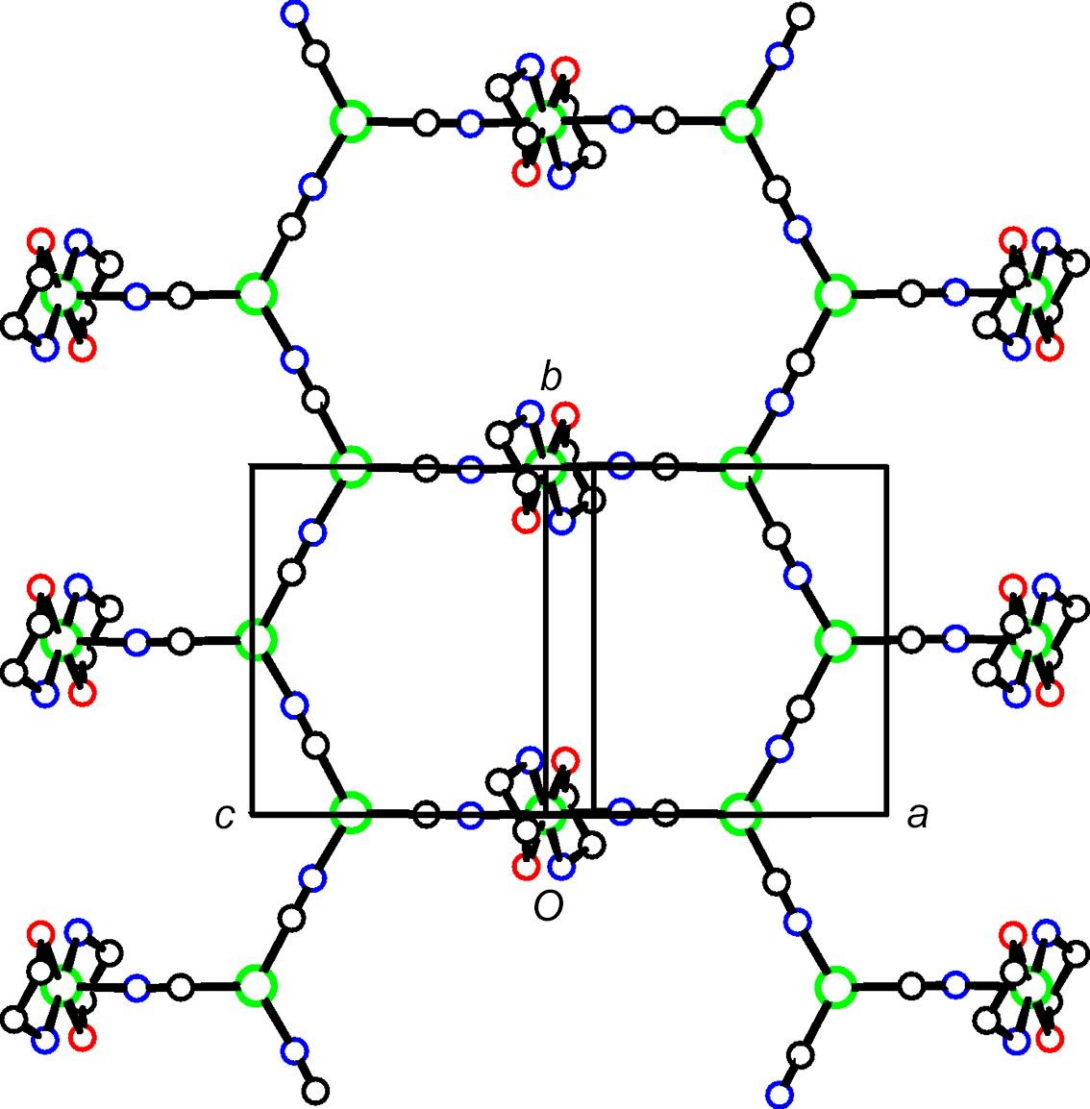
A diperiodic sheet in **3**. The colors are as in Fig. 2 ▸.

The structures of **2** and **3** have the same space group and very similar unit-cell dimensions. In both structures, the Cu2 moieties bridge monoperiodic Cu^I^CN chains to form diperiodic networks parallel to (102), as shown in Figs. 8 ▸ and 9 ▸ for **2**, and in Figs. 10 ▸ and 11 ▸ for **3**. Inversion-related Cu1 atoms from neighboring sheets are 2.7400 (15) Å apart in **2** and 2.7734 (7) Å apart in **3**. In each structure, Cu1 atoms are pulled out of the plane of their three coordinated atoms towards the neighboring inversion-related Cu1 atom, 0.302 (4) Å in **2** and 0.1774 (16) Å in **3**. In **2**, the C2 atom of cyanide C2N2 is also positioned somewhat close to the Cu1 atom of the neighboring sheet, at 2.502 (6) Å, which may indicate that the CN group could be regarded as μ_3_-bonded, with very different Cu—C/N distances, rather than the μ_2_-bonding that we have assumed. A similar situation exists for **3**, although here the Cu—C/N distance to the neighboring sheet is even longer, at 2.686 (3) Å.

Putative hydrogen bonds based on *D*⋯*A* ≤ 3.30 Å and *D*—H⋯*A* > 130° are listed in Tables 3 ▸ and 4 ▸ for com­pounds **2** and **3**, respectively. No contacts in **1** fit these criteria. For both com­pounds **2** and **3**, the NH_2_ group is donor to a screw-related O atom, and one OH disorder com­ponent is donor to a C≡N group.

### Electron spin resonance (ESR)

ESR spectra of the powdered sample materials were re­cor­ded at room temperature using a Bruker EMXnano operating at 9.63 GHz (X-band). For all samples reported here, spectral line shape was unaffected by incident microwave power (*i.e.* no saturable com­ponent of the inhomogeneously broadened line), and the general operating parameters were 0.3 mW (incident power); 2 Gauss (field modulation). Spectra were recorded without using the spectrometer’s digital filter in 0.5 Gauss steps for a 1000 Gauss field sweep; the receiver signal acquisition time per step corresponded to four time constants.

**Figure 12:**
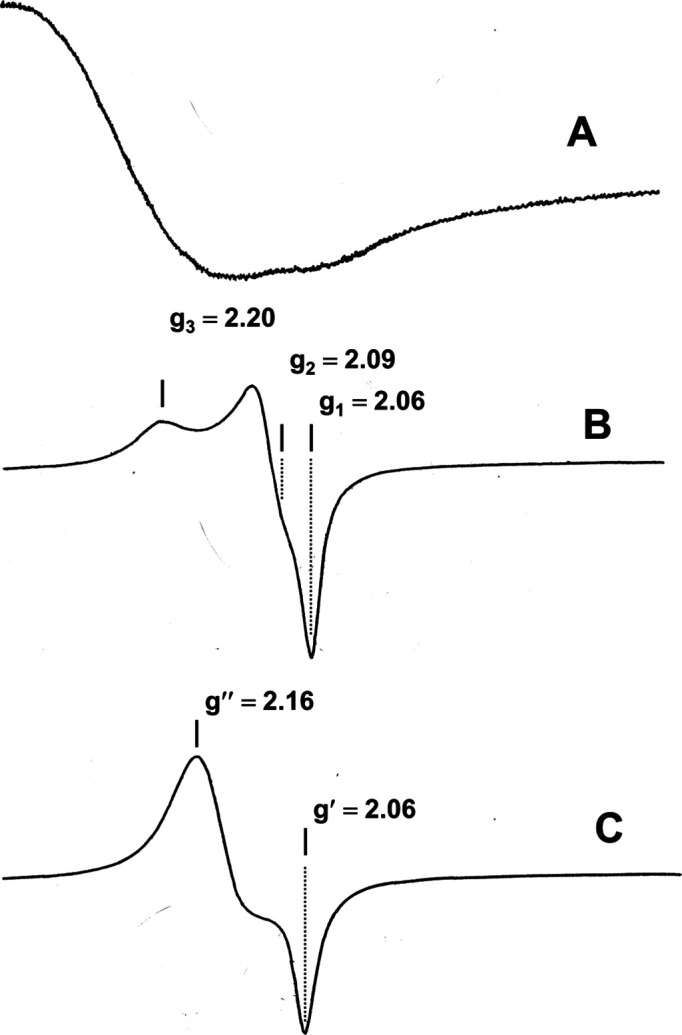
Electron spin resonance (ESR) spectra.

The structure of **1** features two Cu^II^ ion centers bridged by oxygen, and the resultant ESR spectrum is a broad asymmetric singlet with a crossing point at *g* = 2.24 [Fig. 12 ▸(*a*)]. The absence of discernable *g*-anisotropy in the line shape is indicative of spin exchange, as is expected for a bridged binuclear center. The shape and *g*-value determined for this isotropic line is nearly identical to that obtained when a crystal of calcium copper acetate, which ordinarily has a well-defined anisotropic spectrum, is decom­posed at 750 °C; the resulting ESR spectrum of this mixed metal oxide is a broad singlet with *g* = 2.22 (Bender, unpublished).

In contrast to the structure of **1**, the Cu^II^ centers of both structures **2** and **3** are mononuclear, and the corresponding polycrystalline ESR spectra possess line shape features that are rhombic in character (*cf*. Hathaway, 1971 ▸). The turning points in the spectrum of **2** [Fig. 12 ▸(*b*)], corresponding approximately to the diagonal com­ponents of the *g*-tensor, are *g*_1_ = 2.06, *g*_2_ = 2.09, and *g*_3_ = 2.20. The three unequal pairs of coordinate bonds [*i.e.* bond lengths: 2.60 (Cu—O), 2.056 (Cu—N), and 1.967 Å (Cu—NC)], lead us to expect an elongated-rhombic octa­hedral configuration, and our data com­pare favorably with the literature values (*cf*. Hathaway, 1971 ▸).

The ESR spectrum of polycrystalline **3** [Fig. 12 ▸(*c*)] differs from that of structure **2**, presumably due to the reduced Cu—O distance. The shape of the line is rhombic in character, but lacks the added feature [labeled in Fig. 12 ▸(*b*) as ‘*g*_3_’] that is associated with elongation or its counterpart, com­pression (Hathaway, 1971 ▸). The *g*-values determined from the two turning points are *g*′ = 2.06 and *g*′′ = 2.16; the crossing point occurs at *g*′′′= 2.10.

### Thermogravimetric analysis (TGA)

**Figure 13:**
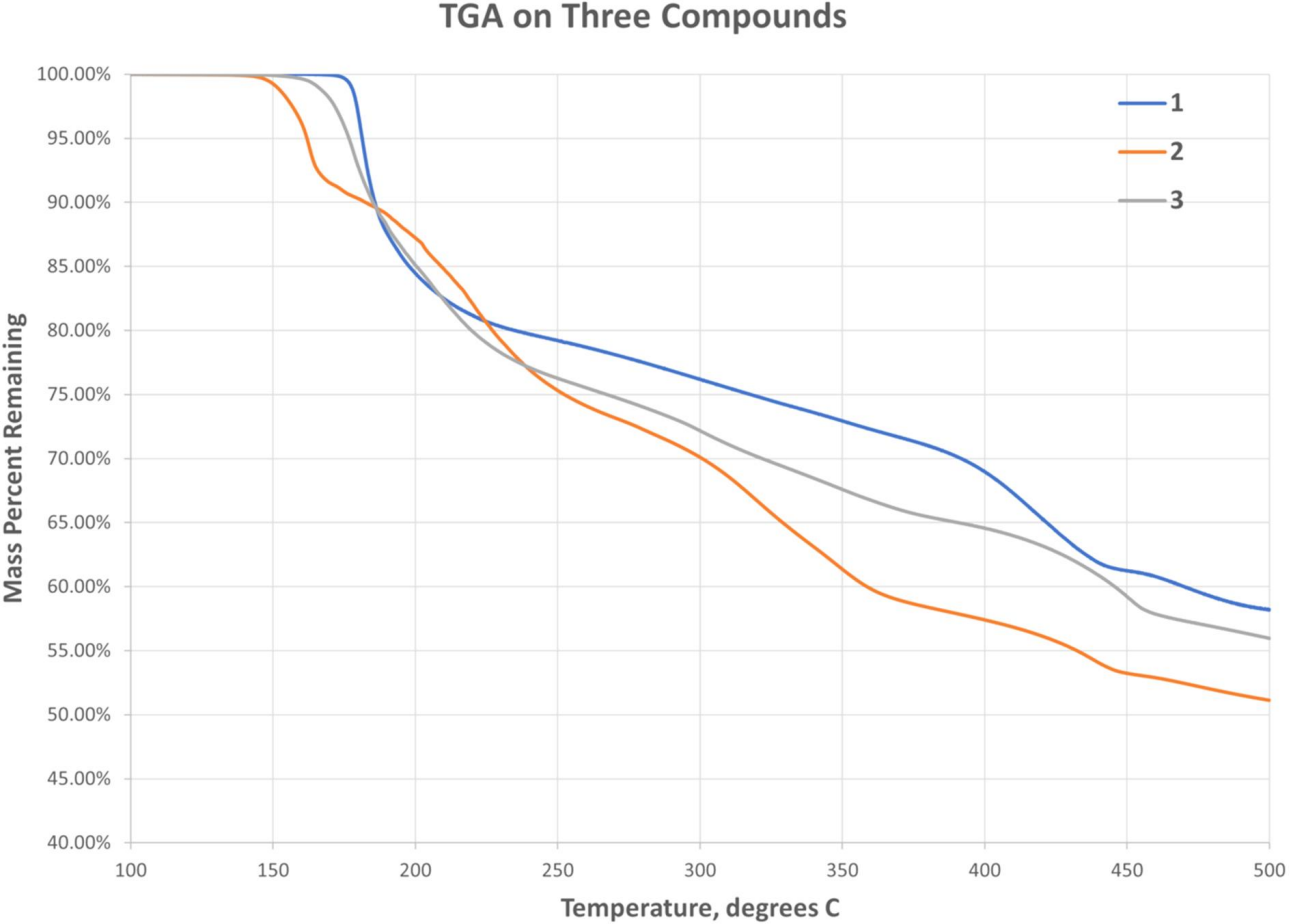
The thermal analysis results for the three com­pounds. The mass percentage remaining is plotted as a function of temperature.

TGA was carried out with a TA Instruments Q500 device. Samples of each com­pound weighing 5–20 mg were heated under nitro­gen gas at 3° min^−1^ to 600 °C or more. The TGA plots up to 500 °C for the three com­pounds are shown in Fig. 13 ▸. (For **1**, analyses were com­plicated, as most samples were heterogeneous. The plot shown is for crystals hand-sorted under the microscope.) In all three com­pounds, there is a sharp drop in mass of 10–15% at 150–200 °C, followed by slower rates of mass loss. The absence of clear steps in the decom­position curves indicates overlapping of incremental decom­position changes. Not shown in the figure are the continued slow mass losses after 500 °C and the occasional subsequent mass increase, presumably due to the formation of an oxide of copper by reaction with residual oxygen in the system. Microscopic examination of residues obtained at the higher temperatures appeared to show mixtures of a black substance and metallic copper.

The decom­position curves for **2** and **3** differ more from each other than might have been expected from the similarity of the structures. In particular, there is a clear change in slope after a mass loss of about 8% for **2**, while the corresponding change is more gradual for **3**, and also the mass differences in the 350–400 °C range are more than would be anti­cipated based upon their mol­ecular formulae.

From previous experiments in our laboratory, we expect any CuCN(s) formed to decom­pose to Cu(s) in the temperature range 400–500 °C. For this reason, each experiment was repeated with termination at 400 °C, in every case leaving black powdery residues. We obtained IR spectra and elemental analyses for each residue at this point, and the results are given in Table 5 ▸. All of the 400 °C residues showed IR peaks indicating the presence of CuCN(s) and, in all cases, the residues were richer in both Cu and C than expected for pure CuCN(s), while showing negligible presence of H. Total percentages ranged from 99.3 to 100.4%, precluding the presence of any significant amount of O. We have inter­preted the residue com­position in terms of mixtures of CuCN(s), C(s), and Cu(s), since it is assumed that any copper(I) acetyl­ide formed would have decom­posed by this temperature, and the percentages calculated from the assumed mixtures are also given in Table 5 ▸. The observed %Cu values were calculated by dividing the %Cu expected from the mol­ecular formula by the fraction of mass remaining at 400 °C. This, of course, assumes that the starting material was pure.

To check for HCN(g) emission at the initial stage, we heated 15–20 mg samples of each of the com­pounds in turn in a test tube, and looked for cloudiness in a drop of AgNO_3_(aq) held over the mouth of the tube. Cloudiness in the AgNO_3_ drop was clearly seen at sand-bath temperatures of 190–200 °C for **2** and 205–215 °C for **3**, but no evidence for HCN(g) emission was noted for **1**, even when the sample was held at or above 250 °C for several minutes.

It is possible that the CN groups bridging Cu1 and Cu2 in **2** and **3** are lost first, combining with the OH protons from the ligands coordinated to Cu2 to form HCN(g), which is not possible in **1**. This would leave the Cu^I^CN chains intact, while the deprotonated ligands would bind to Cu2 more tightly, as they do in **1**. Thereafter, for all three com­pounds, the bound ligands apparently lose their O and N moieties, while leaving at least some of the carbon present in elemental form. Apparently, this begins to happen in **1** before any cyanide loss.

### Database survey

The neutral diperiodic mixed-valence CuCN network of the three structures described here has not often been noted. A search in the Cambridge Structural Database (CSD, Version 5.35; Groom *et al.*, 2016 ▸) found two similar structures (Kim *et al.*, 2005 ▸; Trivedi *et al.*, 2014 ▸). The first involves a CuCN network with Cu^II^ atoms coordinated by cyclam units and the second a more com­plex cyanide/azide network with Cu^II^ coordinated by NH_3_.

A search for Cu com­pounds containing propano­lamine, with or without the OH proton, yielded 48 hits. None of these structures involved Cu bonded to cyanide, however. Of these structures, 44 involved Cu_2_O_2_ units with chelating propano­late chelates, as found in **1**. Only seven of the structures contained the propano­lamine ligand with the OH group intact bonded to Cu, with three of these also containing the Cu_2_O_2_ unit. A separate, more general, search of the CSD for dimeric Cu_2_O_2_ moieties with Cu bonded to two O and two N atoms yielded 258 hits, so that the four-membered ring of Cu_2_O_2_ is not uncommon.

A search for Cu coordinated by 2-aminopro­pan-1-ol gave seven hits. In all but one case (CSD refcode BOYPIO; Nieuwpoort *et al.*, 1983 ▸; Marsh, 2005 ▸), the base coordinates with the OH proton intact, as in Podjed *et al.* (2022 ▸).

A search for Cu coordinated by two ethano­lamine ligands, with or without the OH proton, produced only three examples, *i.e.* Tudor *et al.* (2006 ▸), Vasileva *et al.* (1994 ▸), and the work by Jin *et al.* (2006 ▸) cited earlier.

## Supplementary Material

Crystal structure: contains datablock(s) 1, 2, 3, global. DOI: 10.1107/S2053229624003371/dg3052sup1.cif

Structure factors: contains datablock(s) 1. DOI: 10.1107/S2053229624003371/dg30521sup2.hkl

Structure factors: contains datablock(s) 2. DOI: 10.1107/S2053229624003371/dg30522sup3.hkl

Structure factors: contains datablock(s) 3. DOI: 10.1107/S2053229624003371/dg30523sup4.hkl

CCDC references: 2348772, 2348771, 2348770

## Figures and Tables

**Figure 4:**
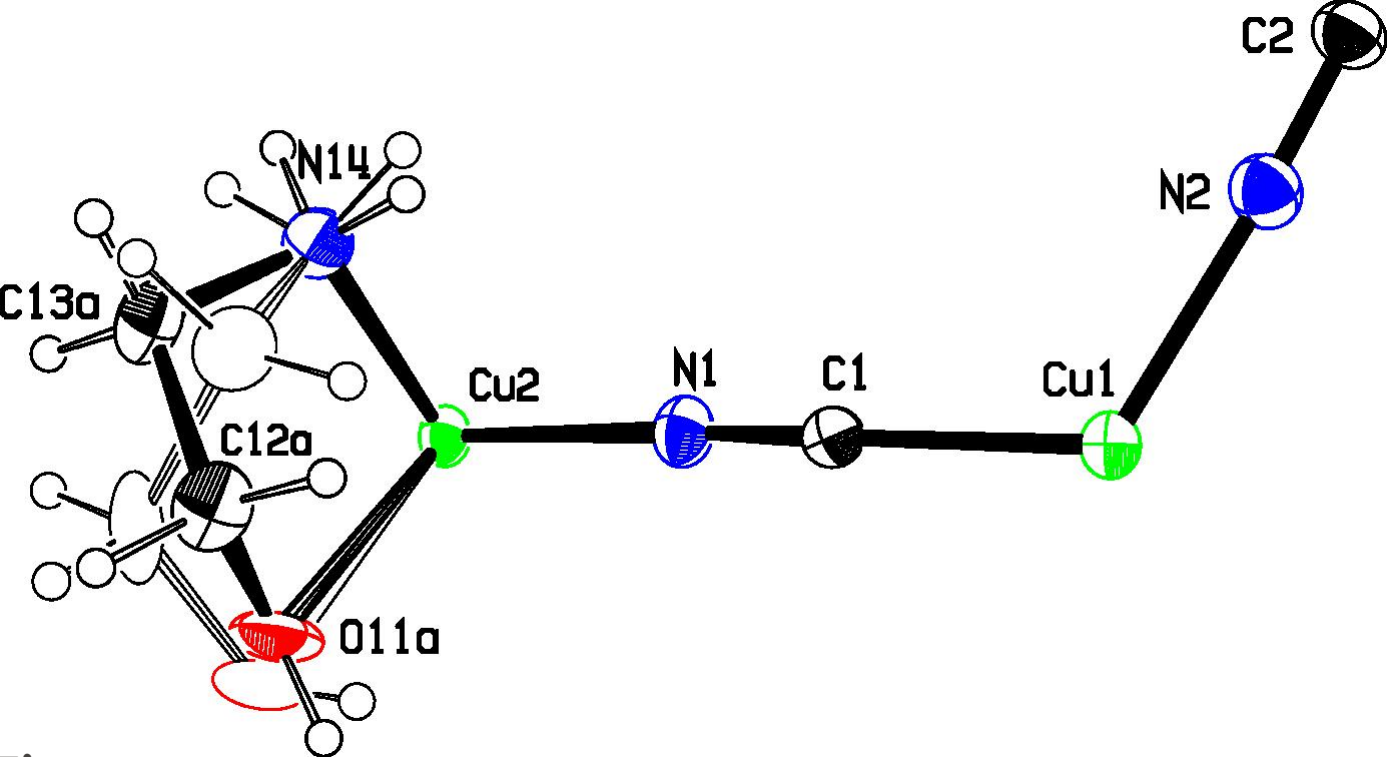
The asymmetric unit of **3**, showing the atom numbering and 50% displacement ellipsoids. The colors are as in Fig. 2 ▸.

**Figure 9:**
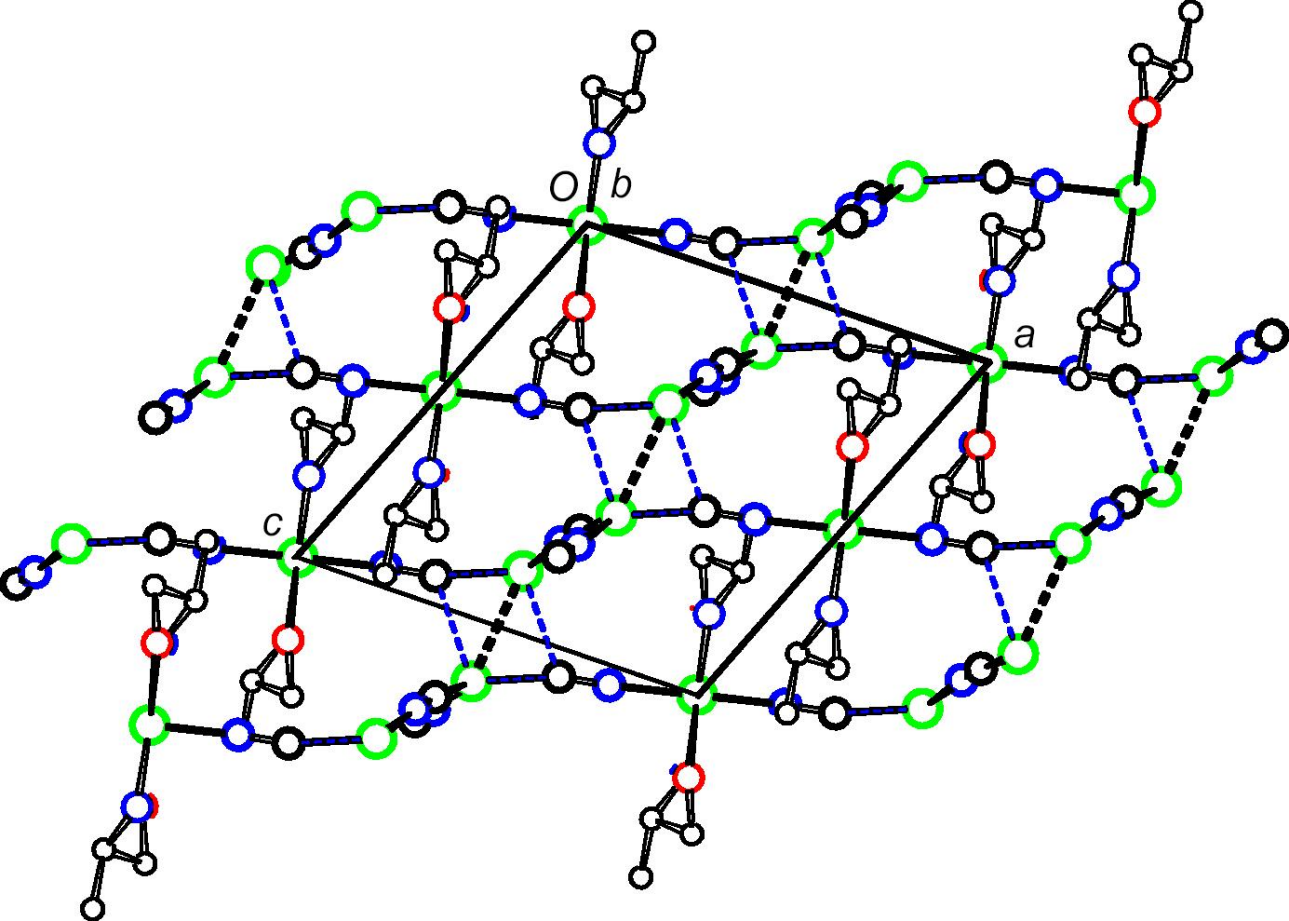
Packing in **2**. The sheets shown in Fig. 8 ▸ are viewed edge on. Putative cuprophilic bonds are shown as dashed double lines and putative μ_3_-C—Cu bonds as blue single-dashed lines.

**Figure 11:**
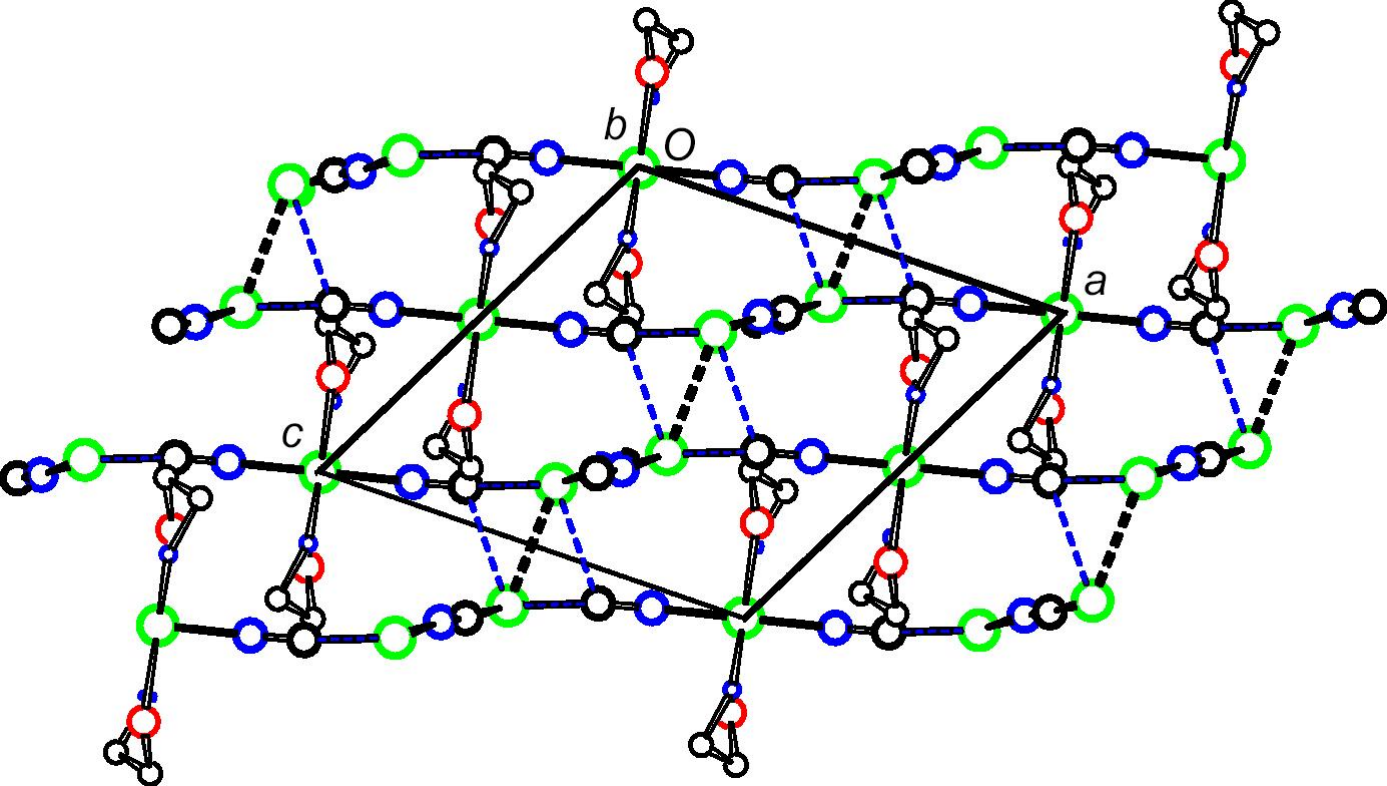
The packing in **3**. The sheets shown in Fig. 10 ▸ are viewed edge on. Putative cuprophilic bonds are shown as dashed double lines and putative μ_3_-C—Cu bonds as blue single-dashed lines.

**Table 1:** Experimental details Experiments were carried out at 295 K with Mo *K*α radiation using an Enraf–Nonius KappaCCD diffractometer. The absorption correction was part of the refinement model (Δ*F*) (Otwinowski & Minor, 1997 ▸). H atoms were treated by a mixture of independent and constrained refinement.

	(1)	(2)	(3)
Crystal data			
Chemical formula	[Cu_4_(CN)_4_(C_3_H_8_NO)_2_]	[Cu_3_(CN)_4_(C_3_H_9_NO)_2_]	[Cu_3_(CN)_4_(C_2_H_7_NO)_2_]
*M*_r_	506.48	444.94	416.88
Crystal system, space group	Monoclinic, *C*2/*c*	Monoclinic, *P*2_1_/*c*	Monoclinic, *P*2_1_/*c*
*a*, *b*, *c* (Å)	9.6829 (4), 8.2557 (4), 21.4992 (10)	9.3903 (4), 8.9608 (4), 9.7986 (4)	9.5158 (2), 8.8022 (2), 9.3589 (2)
β (°)	95.212 (3)	112.134 (3)	117.358 (1)
*V* (Å^3^)	1711.52 (14)	763.74 (6)	696.22 (3)
*Z*	4	2	2
μ (mm^−1^)	4.91	4.15	4.55
Crystal size (mm)	0.31 × 0.15 × 0.08	0.15 × 0.08 × 0.02	0.37 × 0.25 × 0.22

Data collection			
*T*_min_, *T*_max_	0.364, 0.514	0.75, 0.93	0.48, 0.59
No. of measured, independent and observed [*I* > 2σ(*I*)] reflections	19341, 1965, 1501	19192, 1350, 855	27010, 1740, 1049
*R*_int_	0.048	0.084	0.046
(sin θ/λ)_max_ (Å^−1^)	0.649	0.595	0.678

Refinement			
*R*[*F* ^2^ > 2σ(*F* ^2^)], *wR*(*F* ^2^), *S*	0.025, 0.074, 1.09	0.034, 0.113, 1.16	0.024, 0.077, 1.16
No. of reflections	1965	1350	1740
No. of parameters	124	117	118
No. of restraints	0	28	26
Δρ_max_, Δρ_min_ (e Å^−3^)	0.46, −0.42	0.84, −0.48	1.07, −0.53

Computer programs: *KappaCCD Server Software* (Nonius, 1997 ▸), *SCALEPACK* (Otwinowski & Minor, 1997 ▸), *DENZO* (Otwinowski & Minor,1997 ▸), *SHELXT* (Sheldrick, 2015*a* ▸), *SHELXS97* (Sheldrick, 2008 ▸), *SHELXL2018* (Sheldrick, 2015*b* ▸), *ORTEPIII* (Burnett & Johnson, 1996 ▸), *ORTEP-3 for Windows* (Farrugia, 2012 ▸) and *publCIF* (Westrip, 2010 ▸).

**Table 2:** Comparison of selected bond lengths (Å) and angles (°) for **1**, **2** and **3**

	**1**	**2**	**3**
	1.901 (2), 1.923 (2)	2.56 (13), 2.64 (11)	2.439 (14), 2.67 (3)
Cu2—NH_2_	1.976 (3)	2.054 (5)	2.044 (3)
Cu2—NC	1.922 (2)	1.969 (5)	1.963 (2)
C2—Cu1—N2, *trans* to Cu1—C1/N1—Cu2	111.63 (10)	121.7 (2)	120.78 (11)

**Table 3:** Hydrogen-bond geometry (Å, °) for **2** [Chem scheme1]

*D*—H⋯*A*	*D*—H	H⋯*A*	*D*⋯*A*	*D*—H⋯*A*
N14*A*—H14*A*⋯O11*A* ^i^	0.89	2.48	3.21 (11)	140
O11*B*—H11*B*⋯N2^ii^	0.82 (2)	2.54 (13)	3.28 (11)	150 (13)
C12*A*—H12*A*⋯C2N^ii^	0.97	2.54	3.29 (2)	134

Symmetry codes: (i) 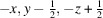 ; (ii) 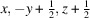 .

**Table 4:** Hydrogen-bond geometry (Å, °) for **3** [Chem scheme1]

*D*—H⋯*A*	*D*—H	H⋯*A*	*D*⋯*A*	*D*—H⋯*A*
N14*B*—H14*D*⋯O11*B* ^i^	0.89	2.20	3.09 (3)	178
O11*B*—H11*B*⋯N2*C* ^ii^	0.82 (1)	2.53 (6)	3.30 (3)	156 (11)

Symmetry codes: (i) 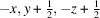 ; (ii) 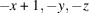 .

**Table 5:** Thermogravimetric analysis data

Compound	**1**	**2**	**3**
Mol­ecular formula	Cu_4_(CN)_4_ *L*′_2_	Cu_3_(CN)_4_ *L*′′_2_	Cu_3_(CN)_4_ *L*′′′_2_
*L*′, *L*′′, *L*′′′	NH_2_(CH_2_)_3_O^−^	NH_2_CH(CH_3_)CH_2_OH	NH_2_CH_2_CH_2_OH
Molar mass, u	506.44	444.98	416.87
% Mass remaining at 400 °C	68.8	57.4	64.6
			
For Residue at 400 °C	Cu, C, H, N	Cu, C, H, N	Cu, C, H, N
Observed Cu, C, H, N %	73.0, 16.0, 0.17, 11.3	75.2, 14.6, 0.24, 9.8	70.8, 15.9, 0.13, 12.6
Calculated Cu, C, H, N %	72.8, 15.7, 0.00, 11.5	76.0, 14.4, 0.00, 9.6	71.3, 16.2, 0.00, 12.6
Assumed com­position for % calculation	5CuCN + 2Cu + 3C	4CuCN + 3Cu + 3C	4CuCN + Cu + 2C
